# Supplementary material for: Approach to Pyrido[2,1-b][1,3]benzothiazol-1-ones via In Situ Generation of Acyl(1,3-benzothiazol-2-yl)ketenes by Thermolysis of Pyrrolo[2,1-c][1,4]benzothiazine-1,2,4-triones
Source: Molecules. 2023 Jul 18;28(14):5495. doi: 10.3390/molecules28145495 (PMC10385387; doi:10.3390/molecules28145495)
Supplement: Supplementary file 1 [file molecules-28-05495-s001.zip › SI_revised.pdf]

Supporting information

for

Approach to Pyrido[2,1-*b*][1,3]benzothiazol-1-ones via *in situ* Generation of Acyl(1,3-benzothiazol-2-yl)ketenes by Thermolysis of Pyrrolo[2,1-*c*][1,4]benzothiazine-1,2,4-triones

Ekaterina A. Lystsova [1], Alexander S. Novikov [2,3], Maksim V. Dmitriev [1], Andrey N. Maslivets [1] and Ekaterina E. Khramtsova [1,*]

[1] Department of Organic Chemistry, Perm State University, ul. Bukireva, 15, 614990 Perm, Russia, caterina.stepanova@psu.ru

[2] Institute of Chemistry, Saint Petersburg State University, Universitetskaya nab. 7/9, 199034 St. Petersburg, Russia

[3] Research Institute of Chemistry, Peoples' Friendship University of Russia (RUDN University), Miklukho-Maklaya Street, 6, Moscow, 117198, Russia

***** Correspondence: caterina.stepanova@psu.ru

Table of contents

NMR charts of compounds **2a-g**…………………………………………………….S3
STA charts of compounds **1a-g**, **4**………………………………….…………...S18
Calculated total electronic energies (E, in Hartree), enthalpies (H, in Hartree),
Gibbs free energies (G, in Hartree), and entropies (S, cal/mol•K) for
optimized equilibrium model structures…………………………………………S26
HPLC data for thermal decomposition of compounds **1a** and **4**………………...S27
ORTEP images of X-ray crystal structures of compounds **2a, f**…………………S30

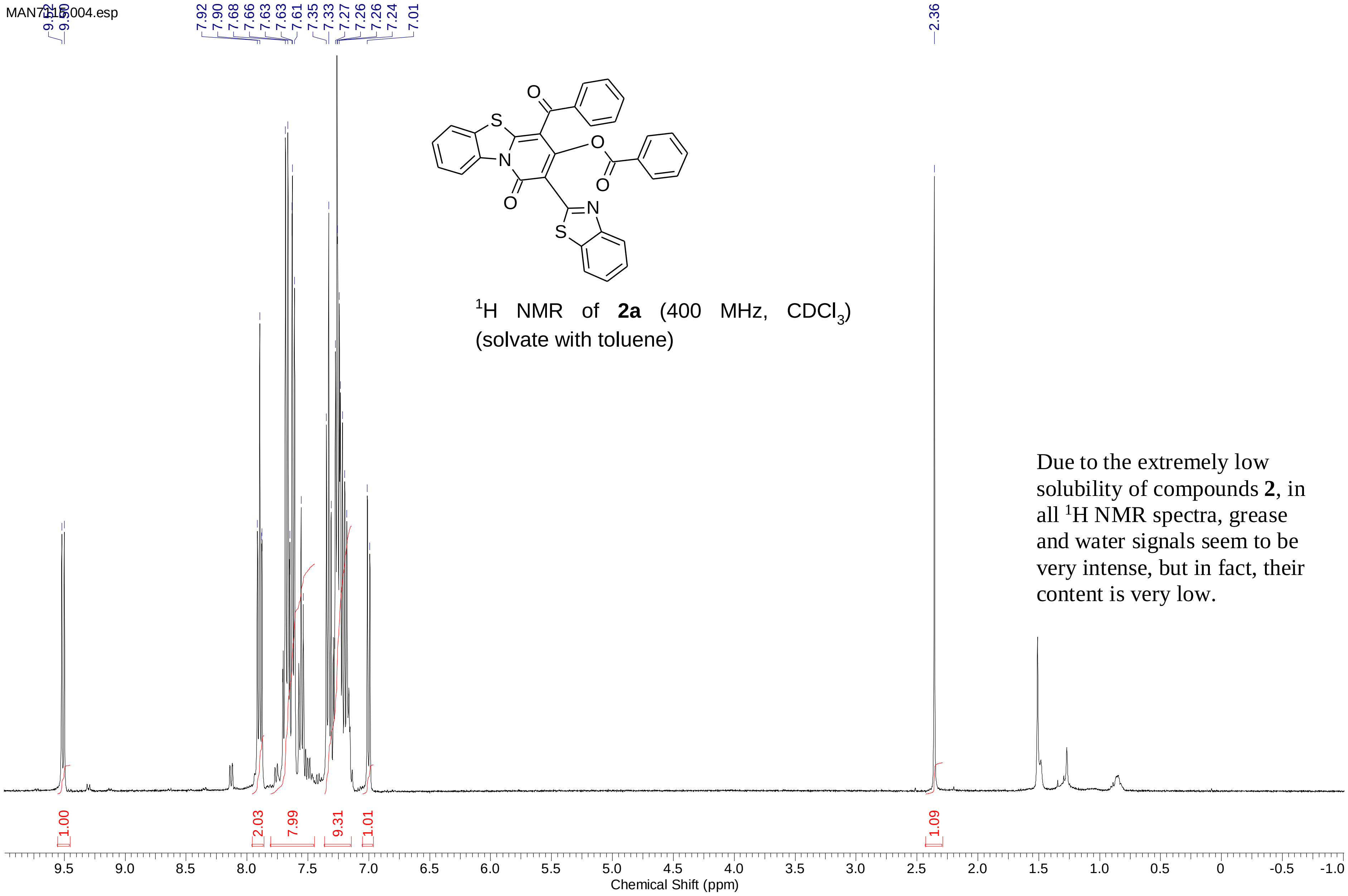

^{1}H NMR of **2a** (400 MHz, $CDCl_3$) (solvate with toluene)

Due to the extremely low solubility of compounds **2**, in all ^{1}H NMR spectra, grease and water signals seem to be very intense, but in fact, their content is very low.

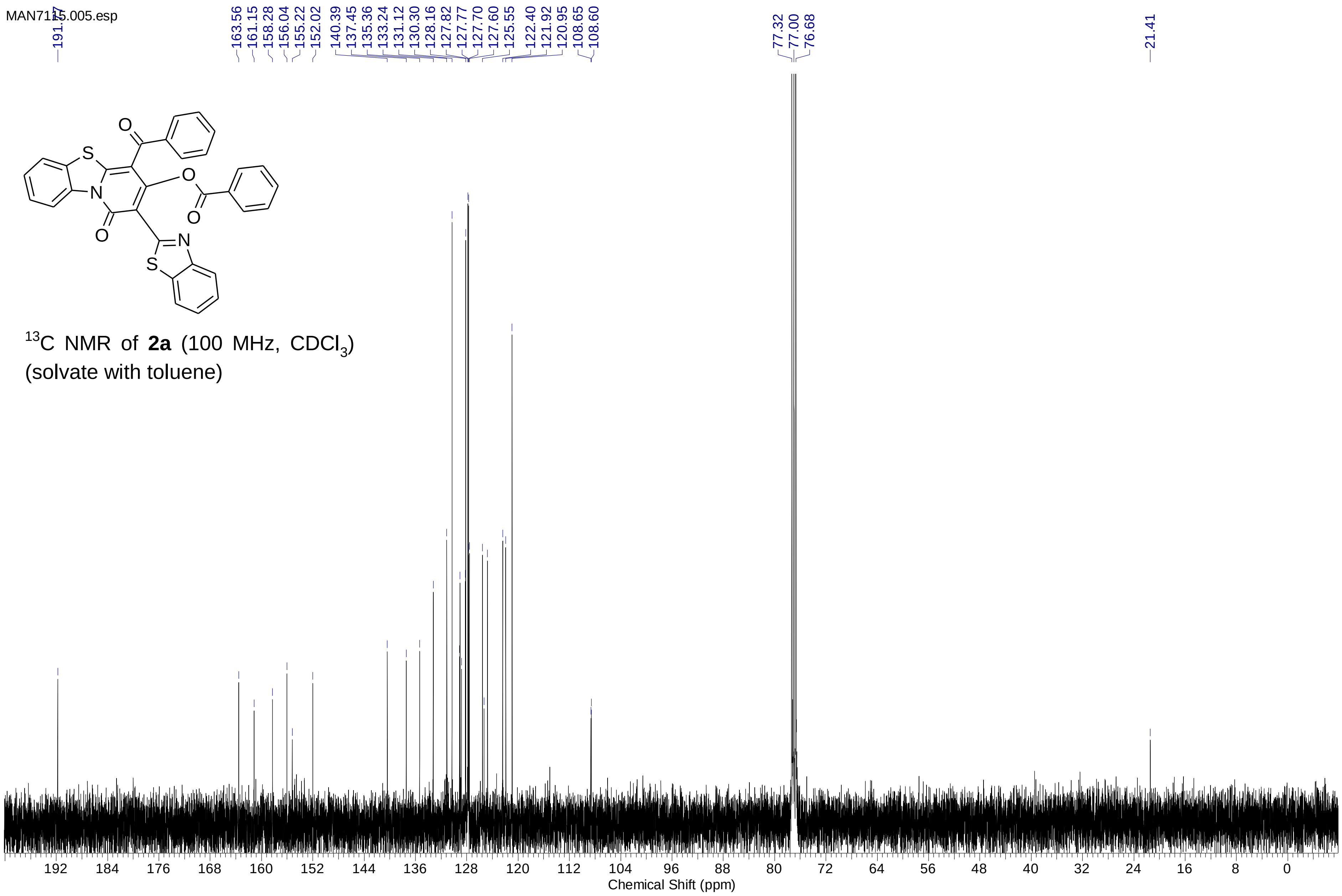

^{13}C NMR of **2a** (100 MHz, $CDCl_3$)
(solvate with toluene)

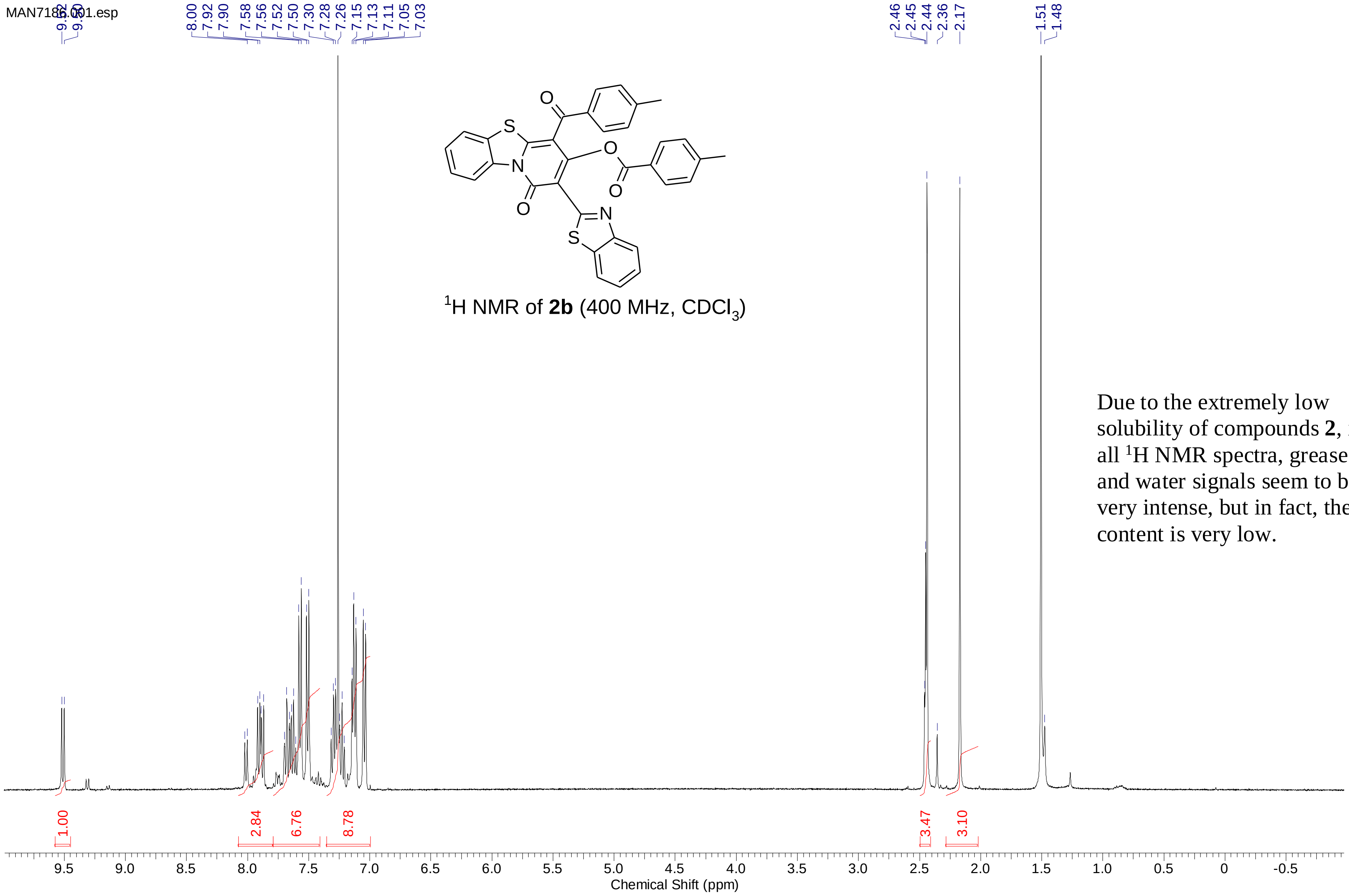

^{1}H NMR of **2b** (400 MHz, $CDCl_3$)

Due to the extremely low solubility of compounds **2**, in all ^{1}H NMR spectra, grease and water signals seem to be very intense, but in fact, their content is very low.

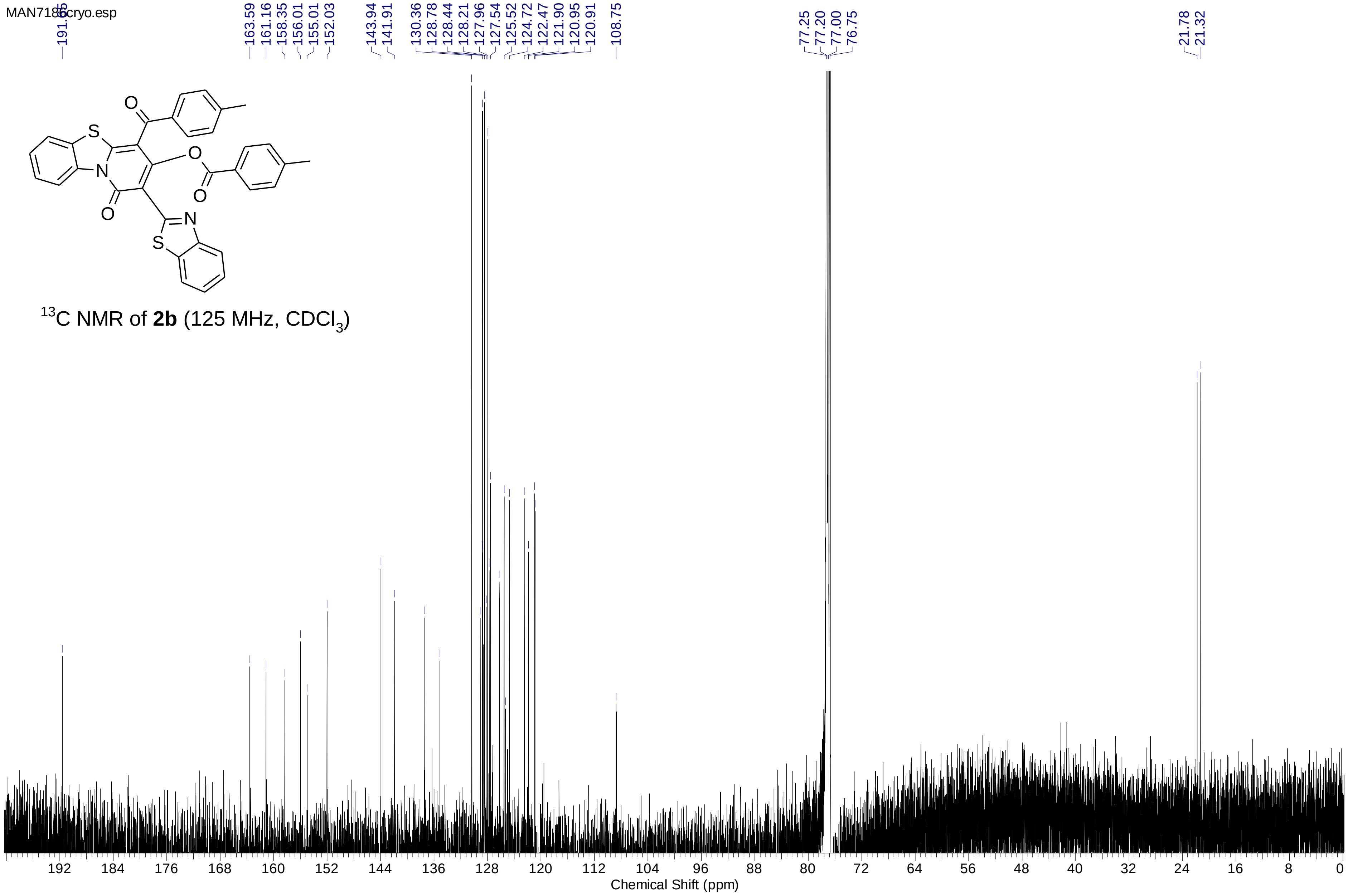

^{13}C NMR of **2b** (125 MHz, $CDCl_3$)

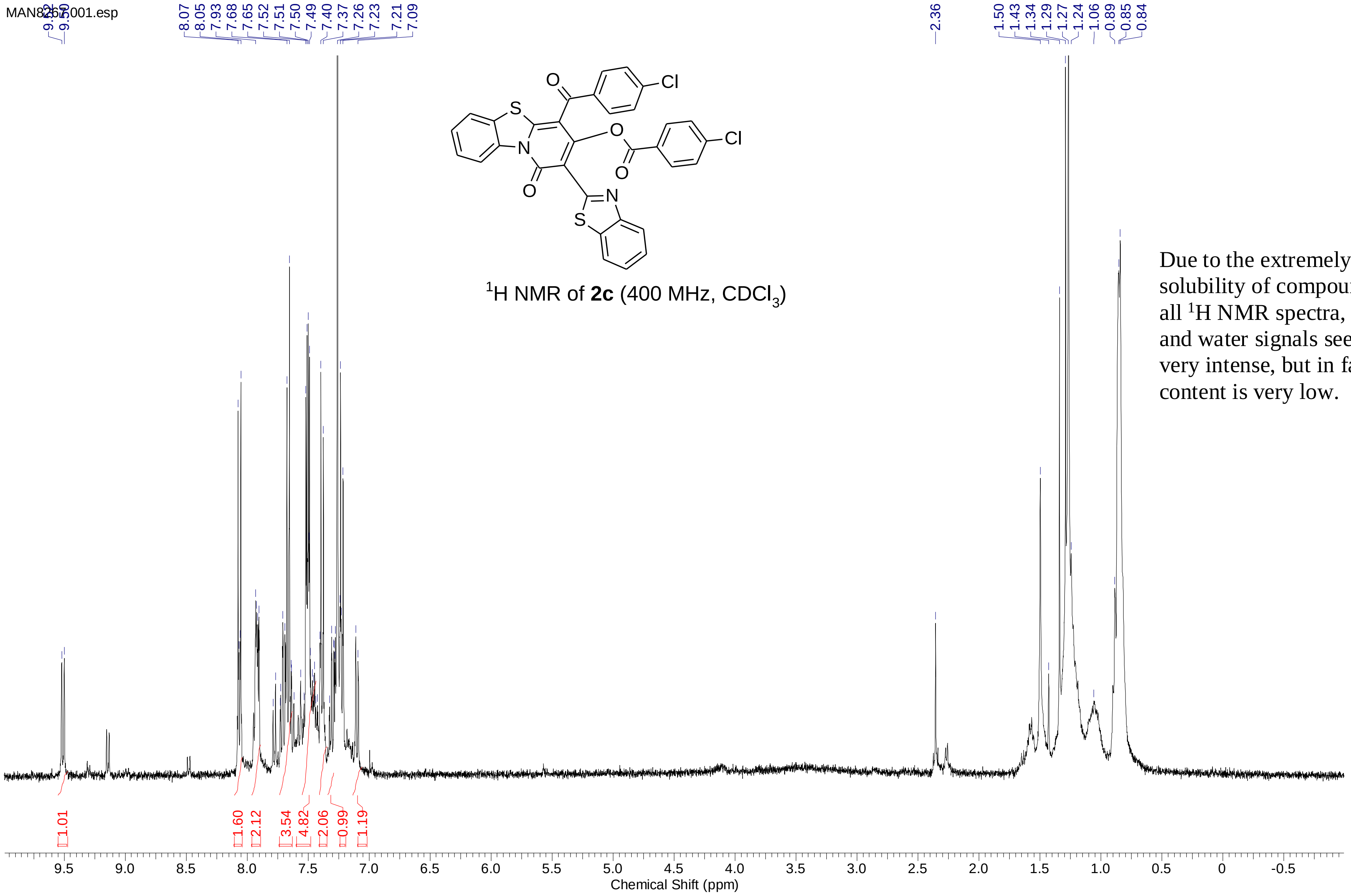

^{1}H NMR of **2c** (400 MHz, $CDCl_3$)

Due to the extremely low solubility of compounds **2**, in all ^{1}H NMR spectra, grease and water signals seem to be very intense, but in fact, their content is very low.

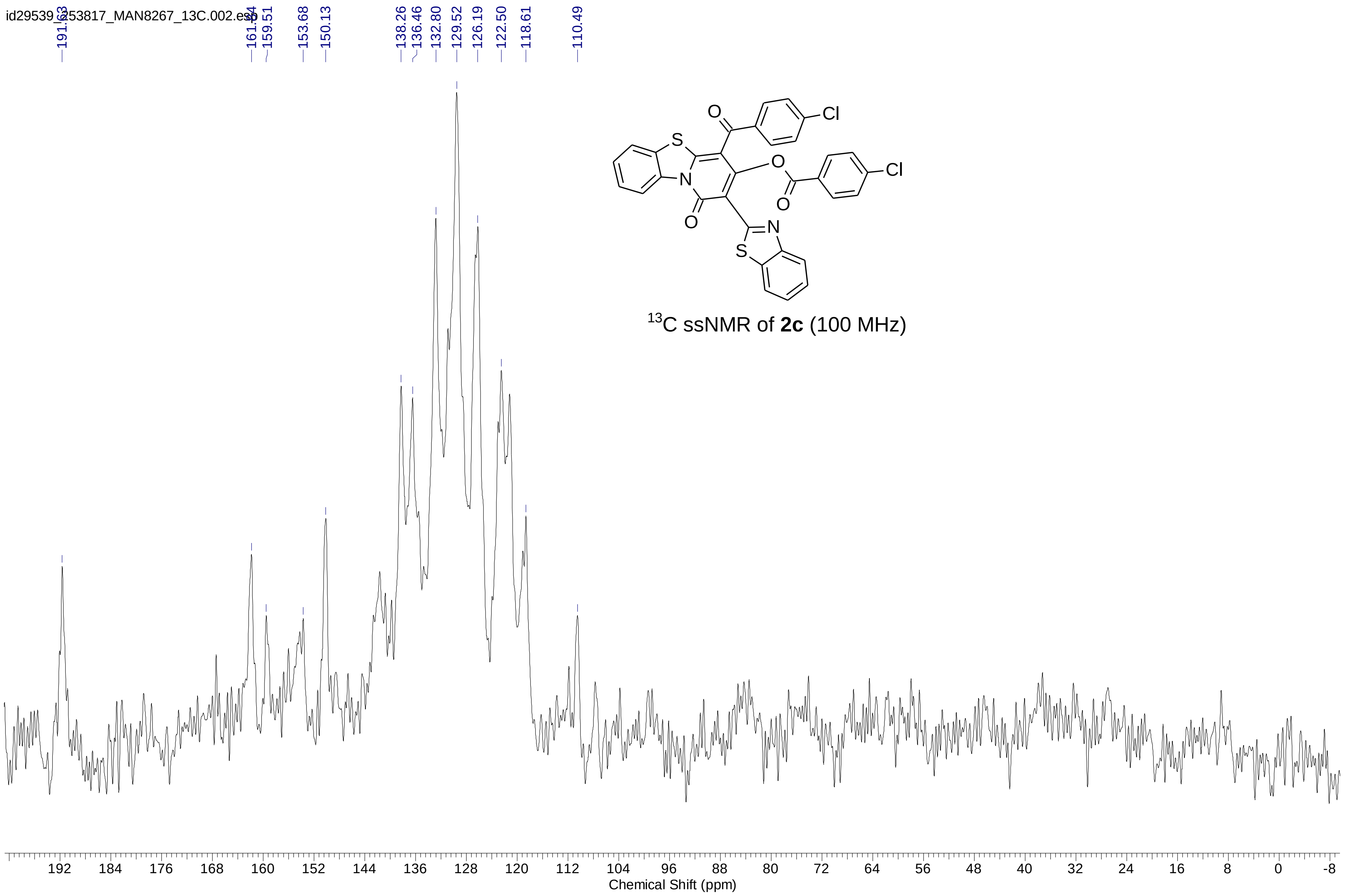

^{13}C ssNMR of **2c** (100 MHz)

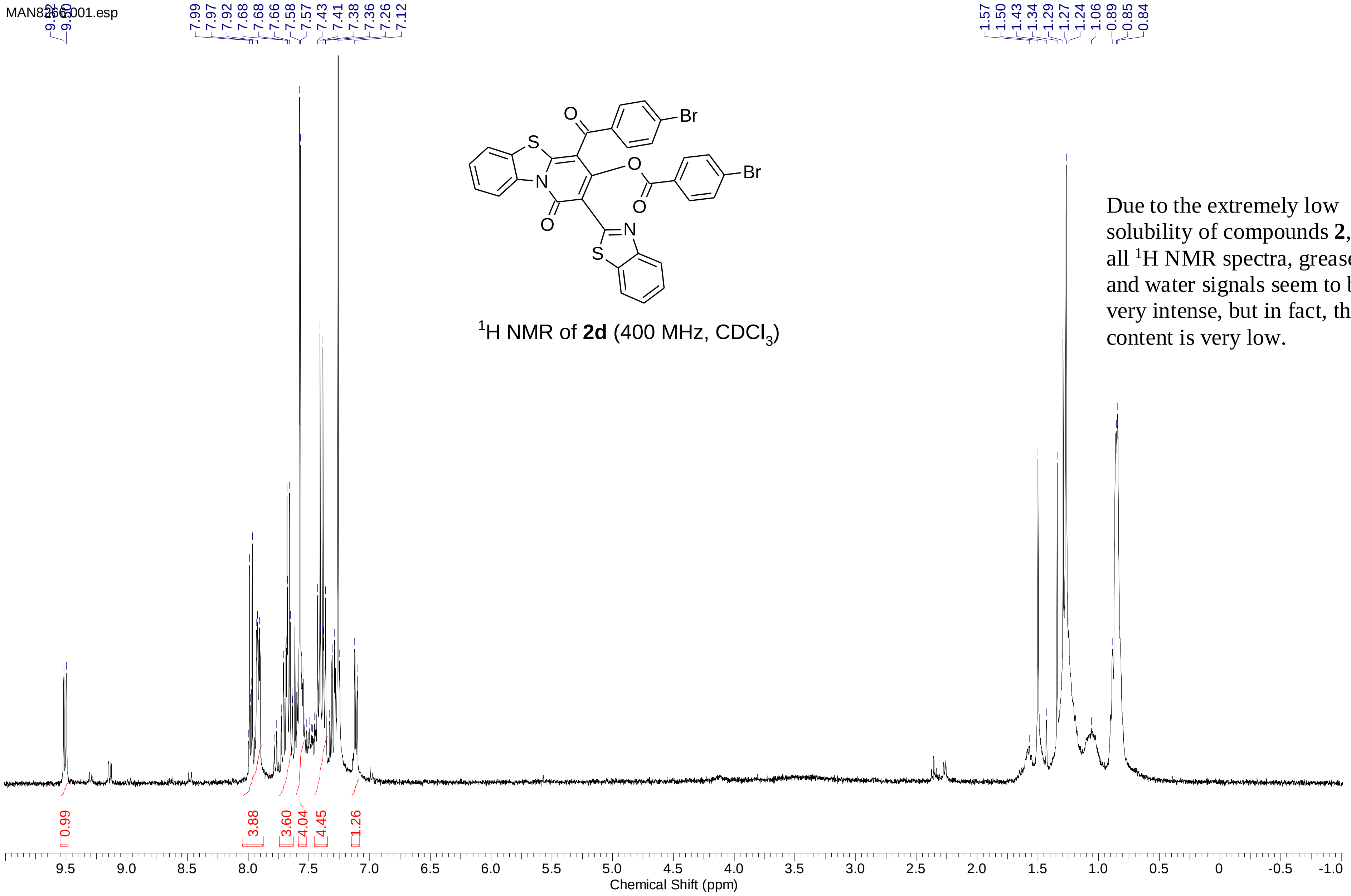

^{1}H NMR of **2d** (400 MHz, $CDCl_3$)

Due to the extremely low solubility of compounds **2**, in all ^{1}H NMR spectra, grease and water signals seem to be very intense, but in fact, their content is very low.

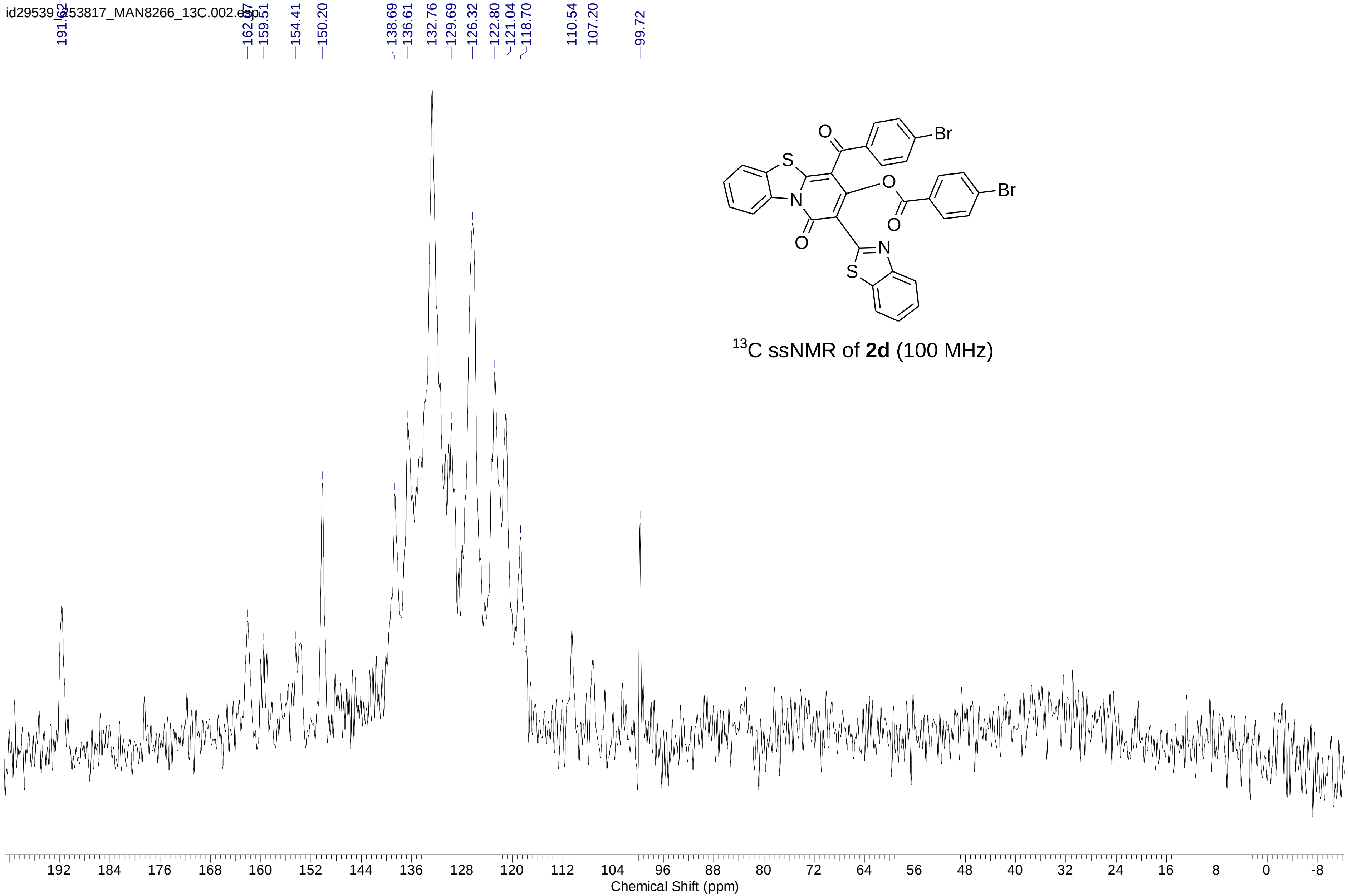

^{13}C ssNMR of **2d** (100 MHz)

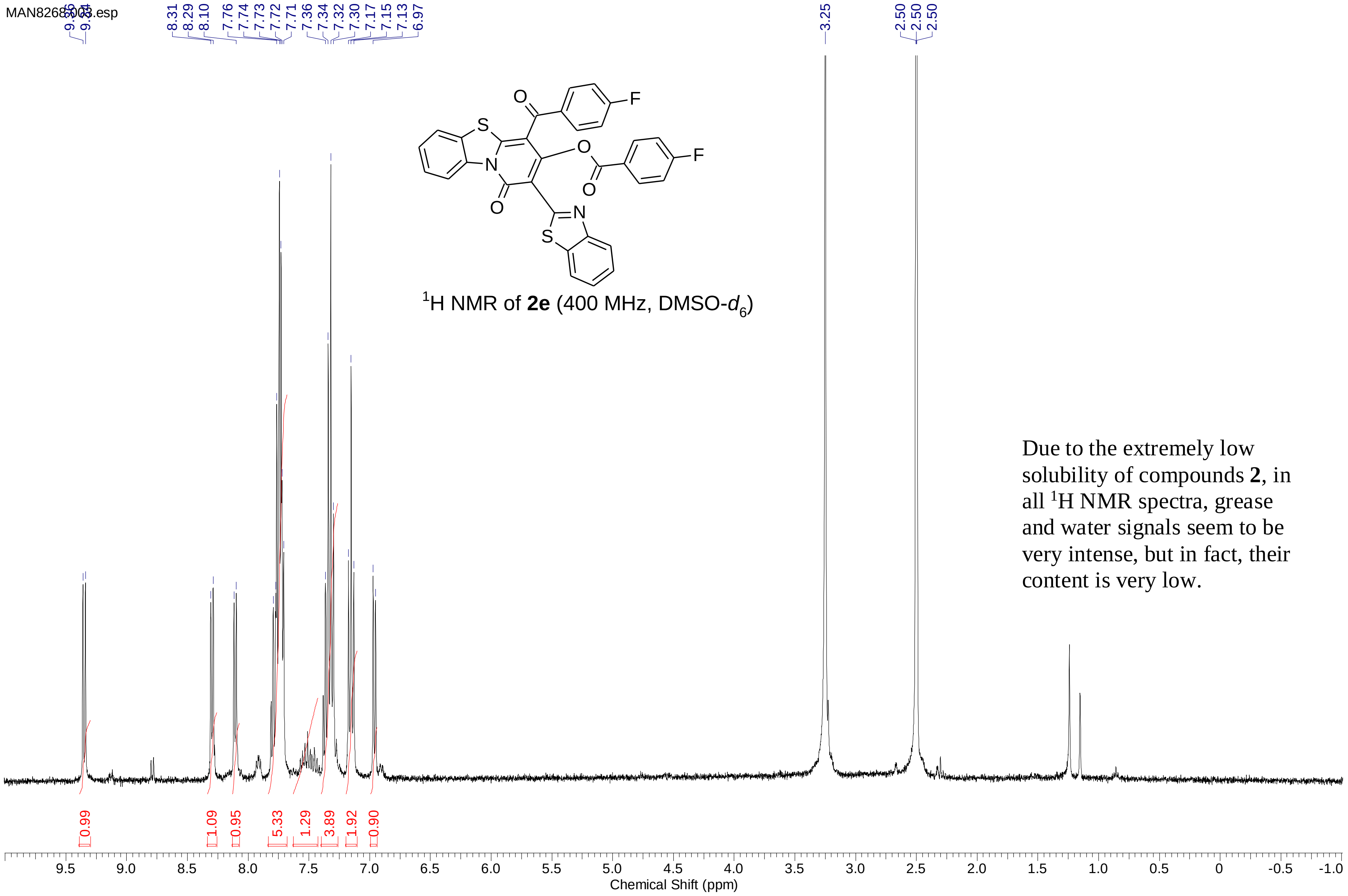

^{1}H NMR of **2e** (400 MHz, DMSO-d_6)

Due to the extremely low solubility of compounds **2**, in all ^{1}H NMR spectra, grease and water signals seem to be very intense, but in fact, their content is very low.

MAN8268.005.esp

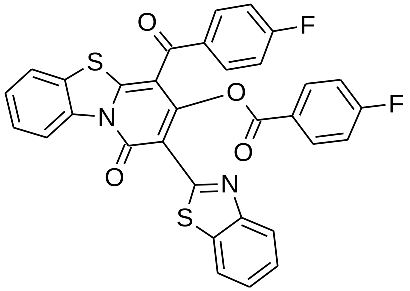

^{19}F NMR of **2e** (376 MHz, DMSO-d_6)

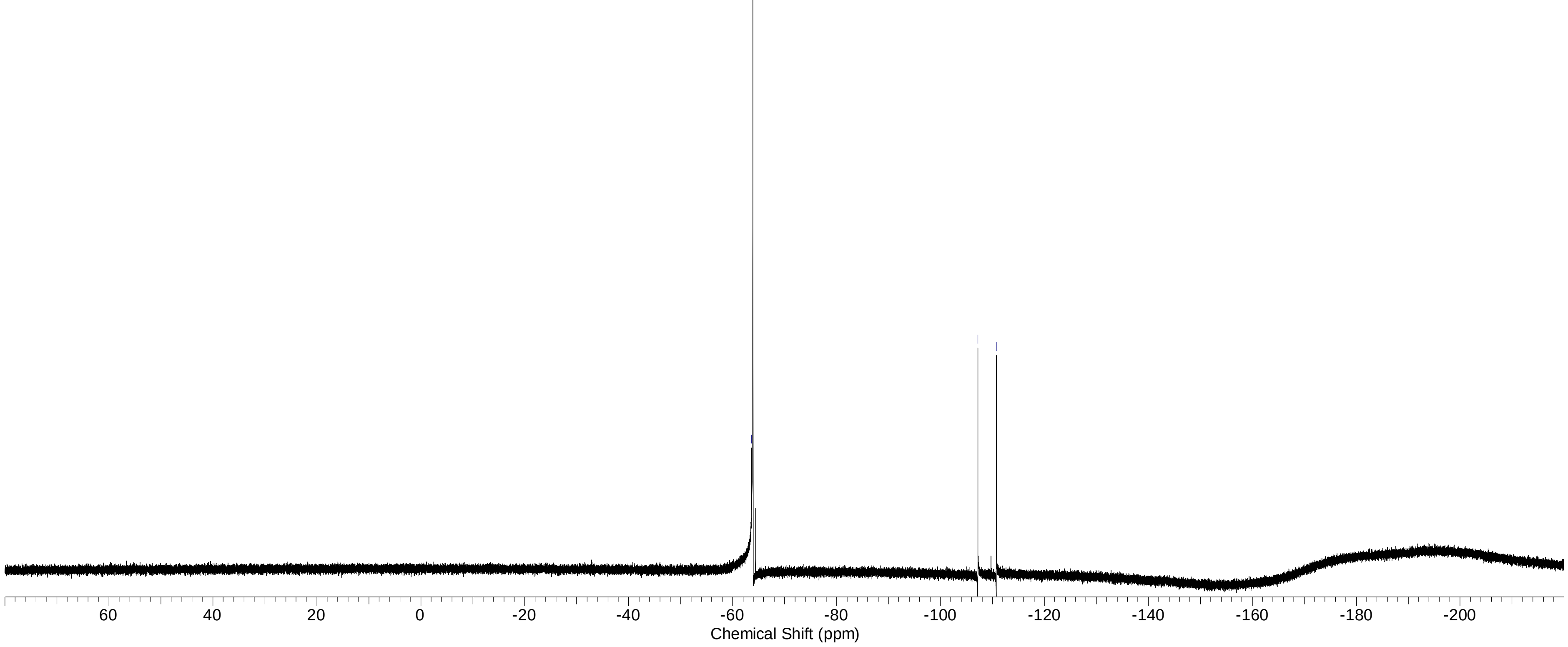

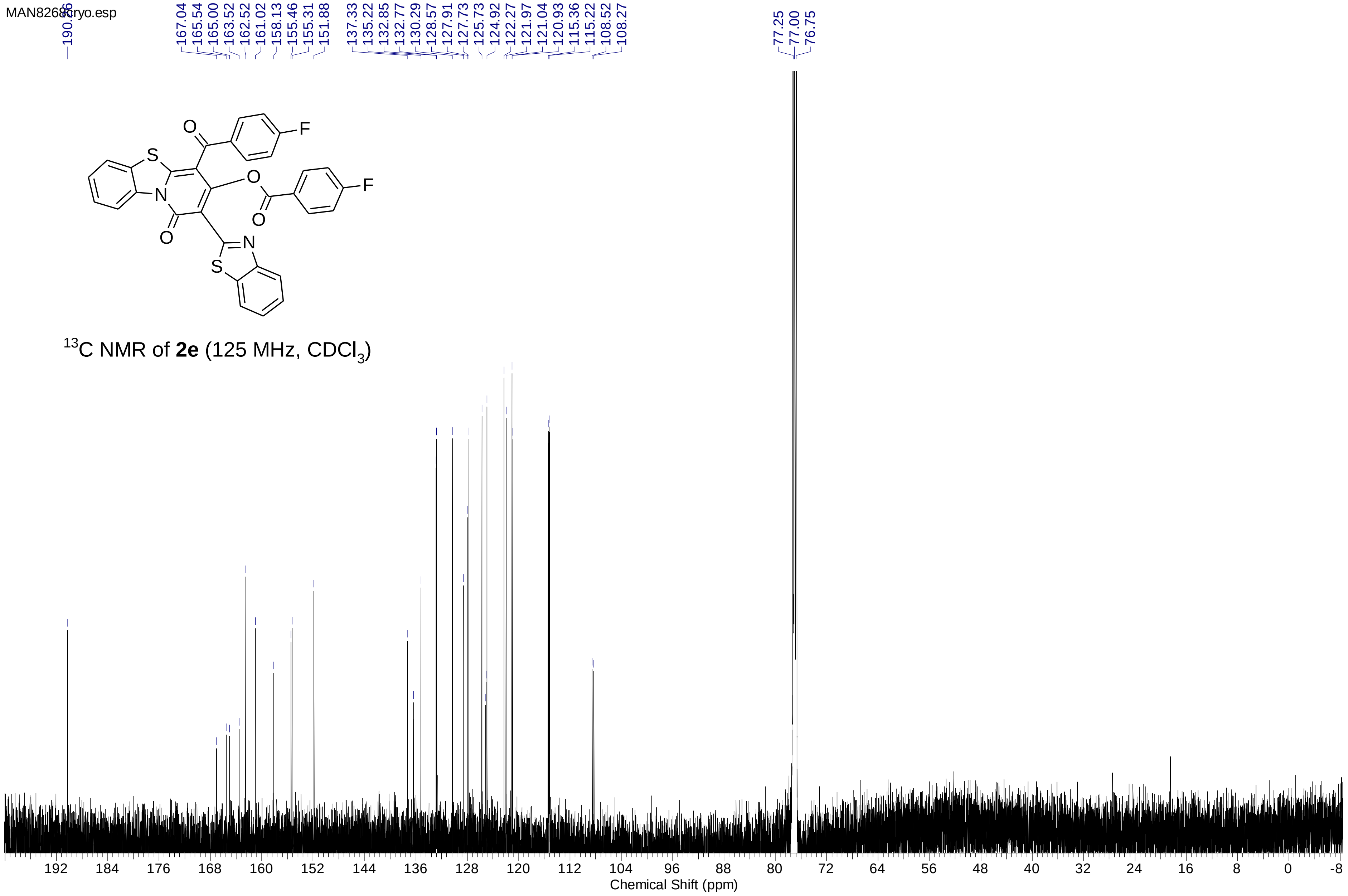

^{13}C NMR of **2e** (125 MHz, $CDCl_3$)

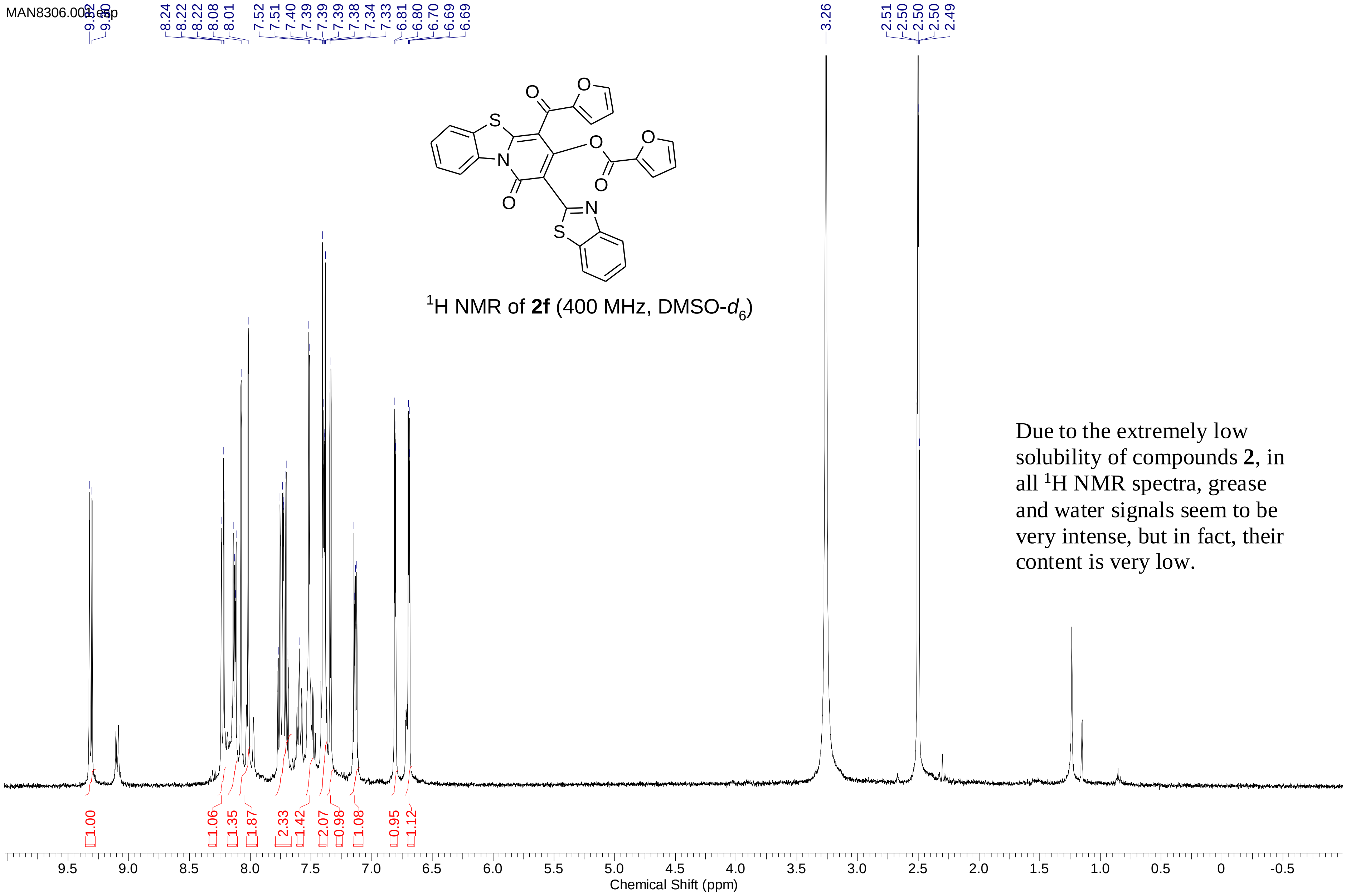

^{1}H NMR of **2f** (400 MHz, DMSO-d_6)

Due to the extremely low solubility of compounds **2**, in all ^{1}H NMR spectra, grease and water signals seem to be very intense, but in fact, their content is very low.

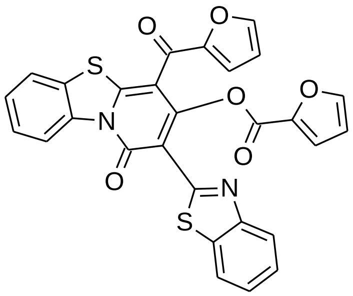

^{13}C NMR of **2f** (125 MHz, $CDCl_3$)

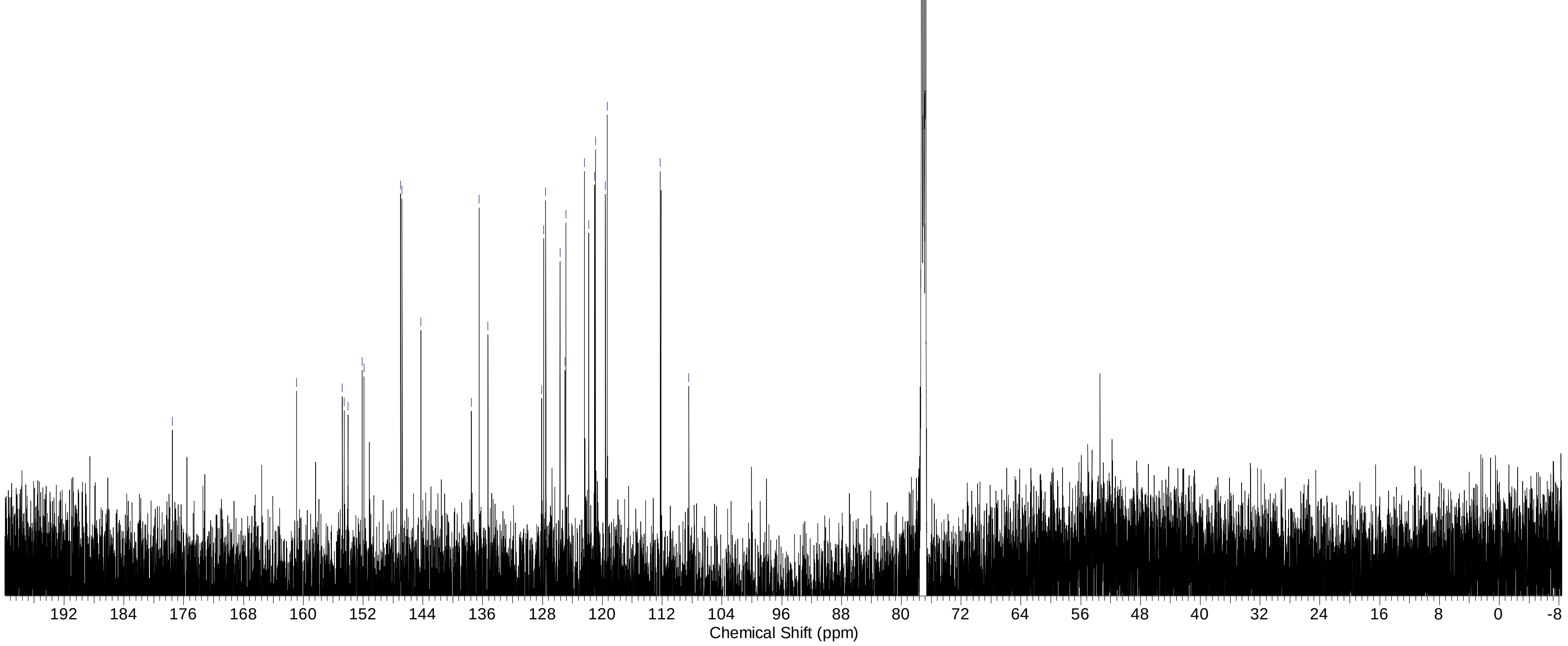

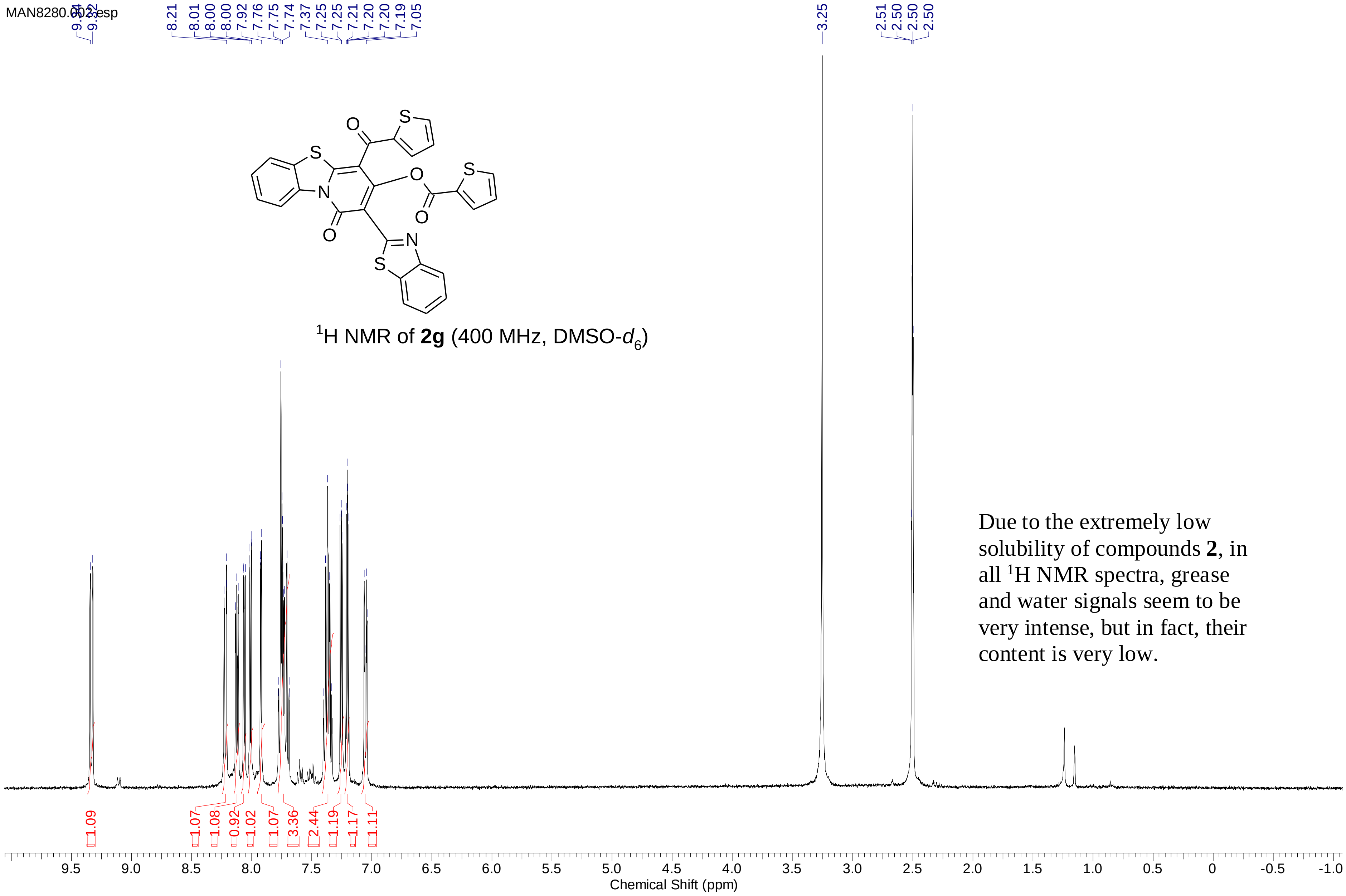

^{1}H NMR of **2g** (400 MHz, DMSO-d_6)

Due to the extremely low solubility of compounds **2**, in all ^{1}H NMR spectra, grease and water signals seem to be very intense, but in fact, their content is very low.

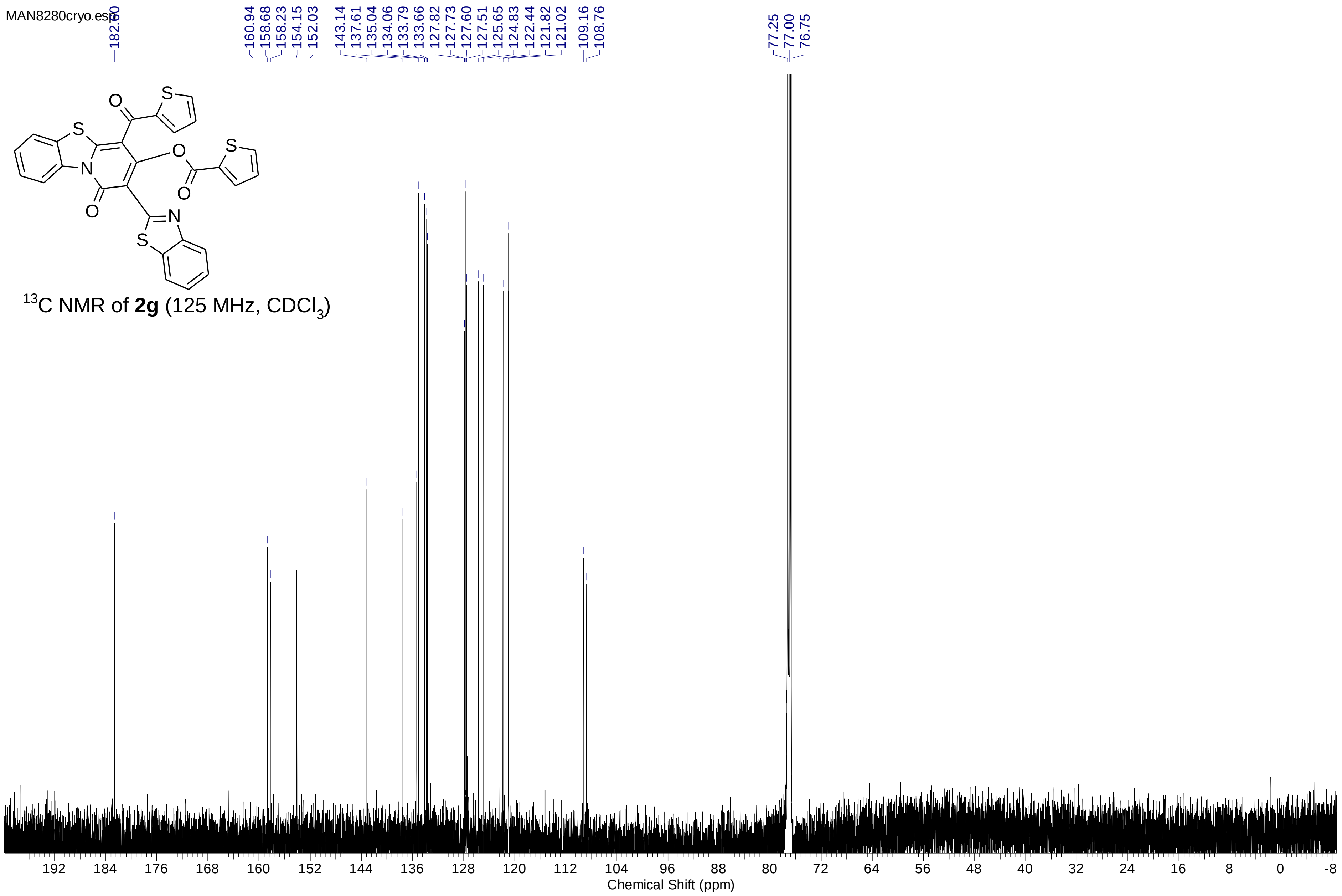

^{13}C NMR of **2g** (125 MHz, $CDCl_3$)

STA for compound **1a** is given in the main manuscript as Figure 2.

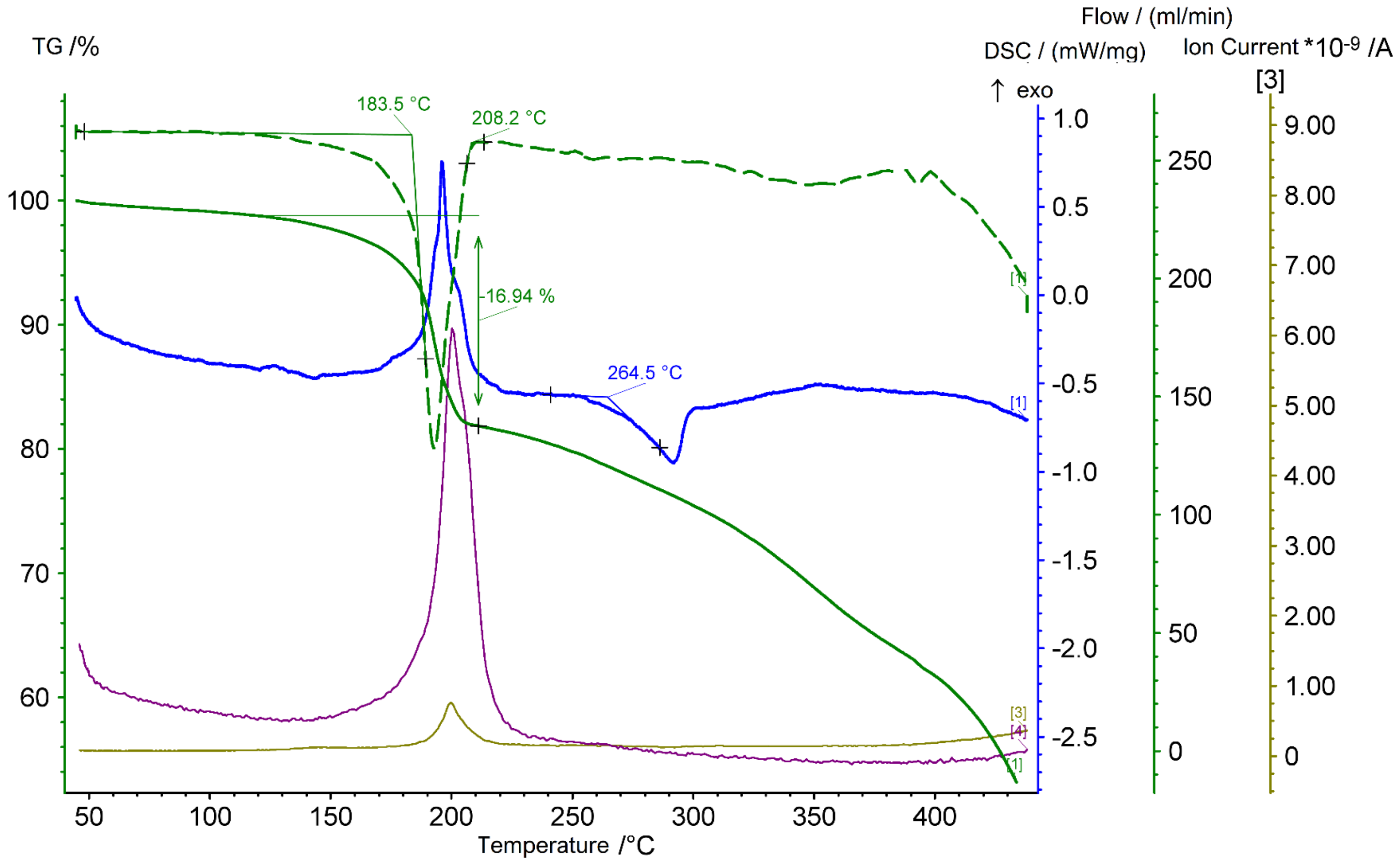

STA-MS plot of thermolysis of APBTT **1b**. Blue solid curve: differential scanning calorimetry (DSC); green solid curve: thermogravimetry (TG); greed dashed curve: derivative thermogravimetry (dTG); violet solid curve: m/z = 28 (MS); brown solid curve: m/z = 44 (MS); heating rate: 10 K/min; argon atmosphere.

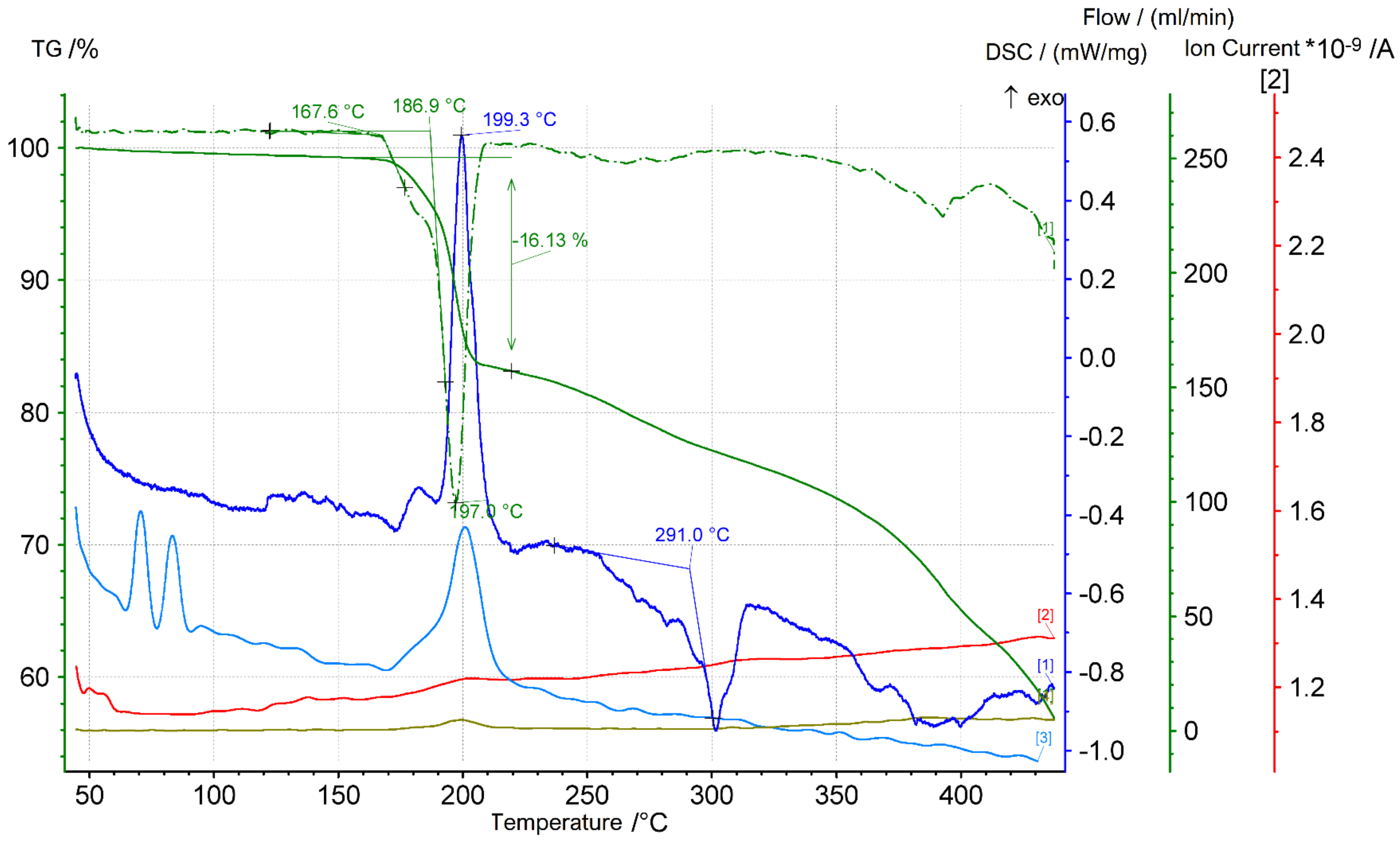

STA-MS plot of thermolysis of APBTT **1c**. Dark blue solid curve: differential scanning calorimetry (DSC); green solid curve: thermogravimetry (TG); greed dashed curve: derivative thermogravimetry (dTG); light blue solid curve: m/z = 28 (MS); brown solid curve: m/z = 44 (MS); red solid curve: m/z = 18 (MS); heating rate: 10 K/min; argon atmosphere.

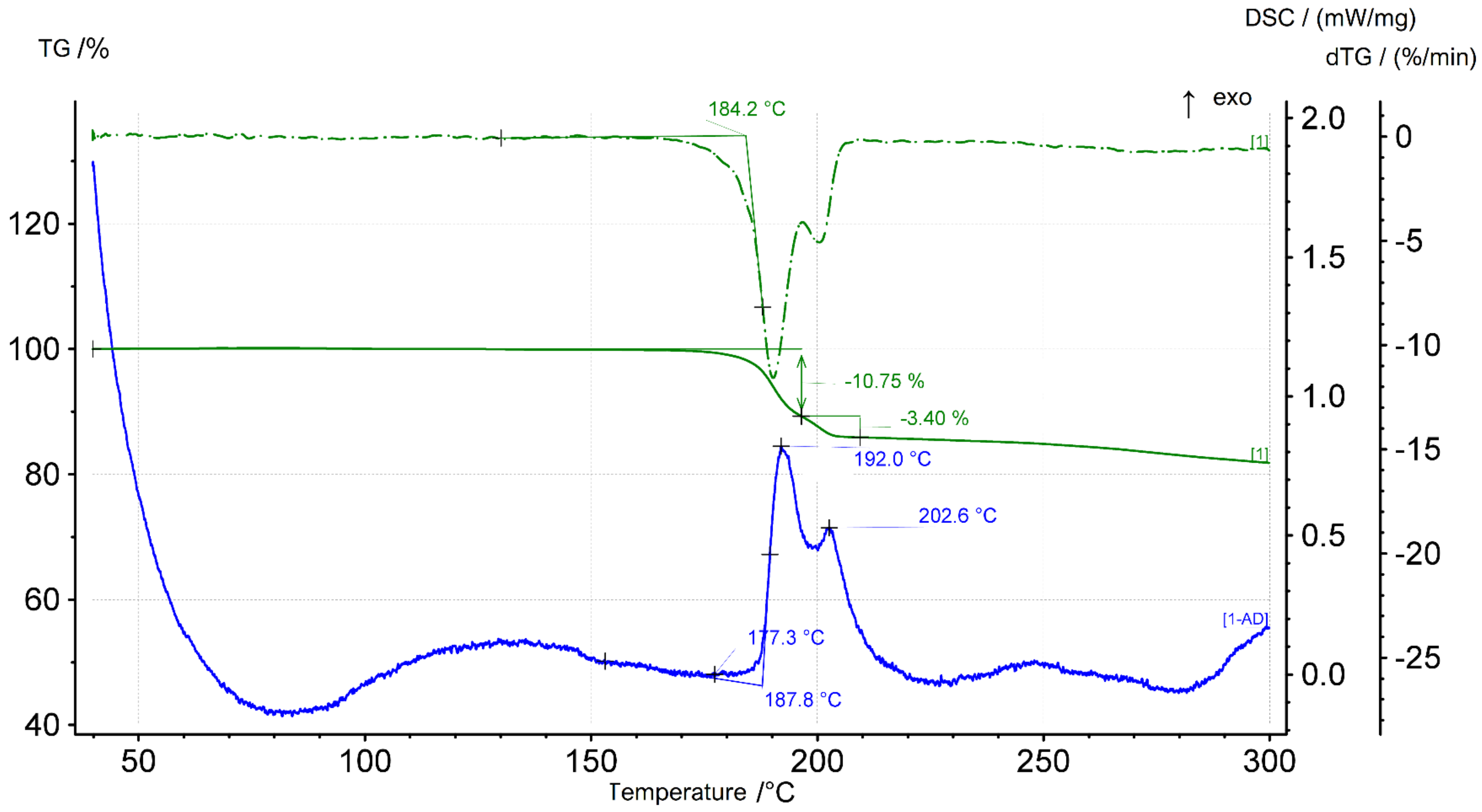

STA plot of thermolysis of APBTT **1d**. Blue solid curve: differential scanning calorimetry (DSC); green solid curve: thermogravimetry (TG); greed dashed curve: derivative thermogravimetry (dTG); heating rate: 10 K/min; argon atmosphere.

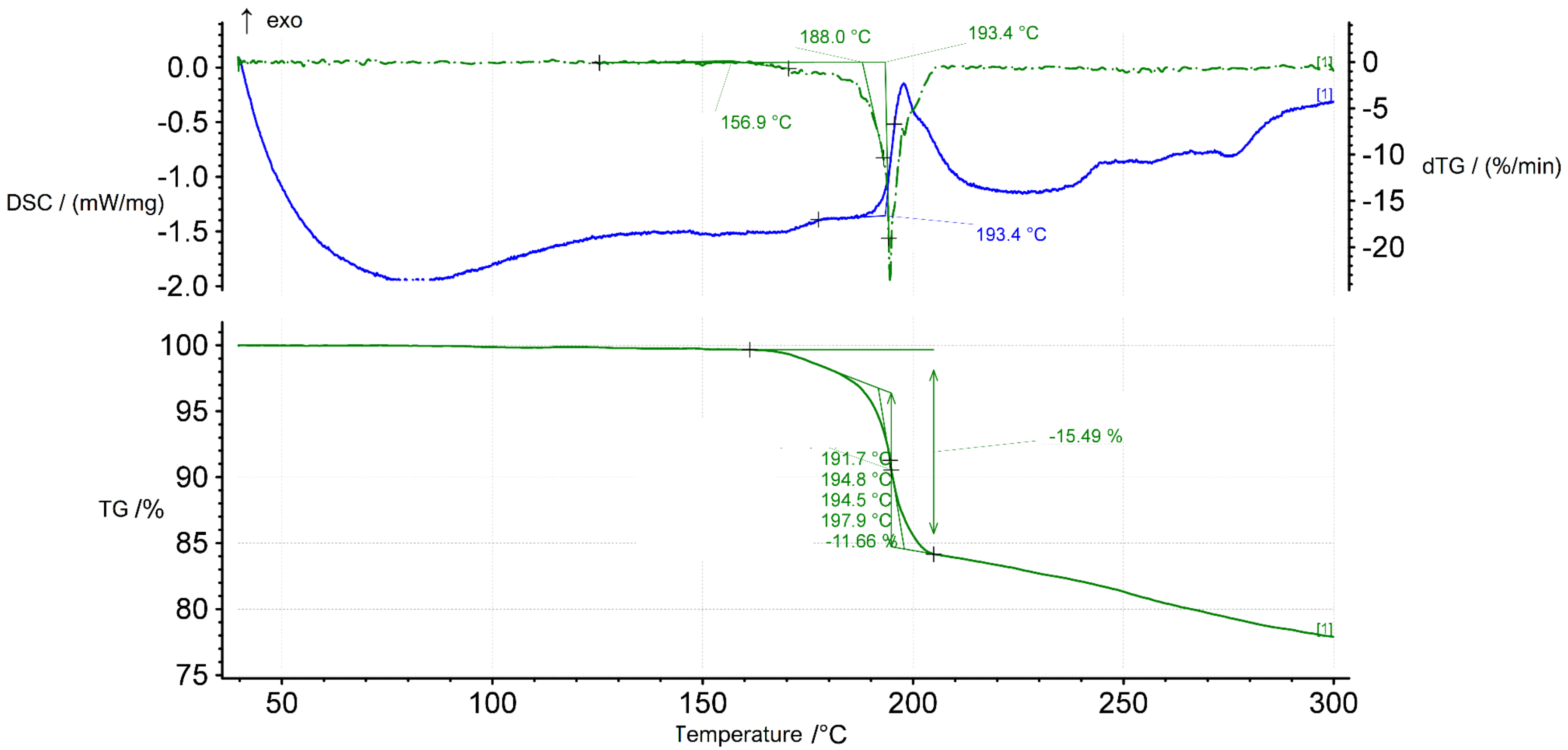

STA plot of thermolysis of APBTT **1e**. Blue solid curve: differential scanning calorimetry (DSC); green solid curve: thermogravimetry (TG); greed dashed curve: derivative thermogravimetry (dTG); heating rate: 10 K/min; argon atmosphere.

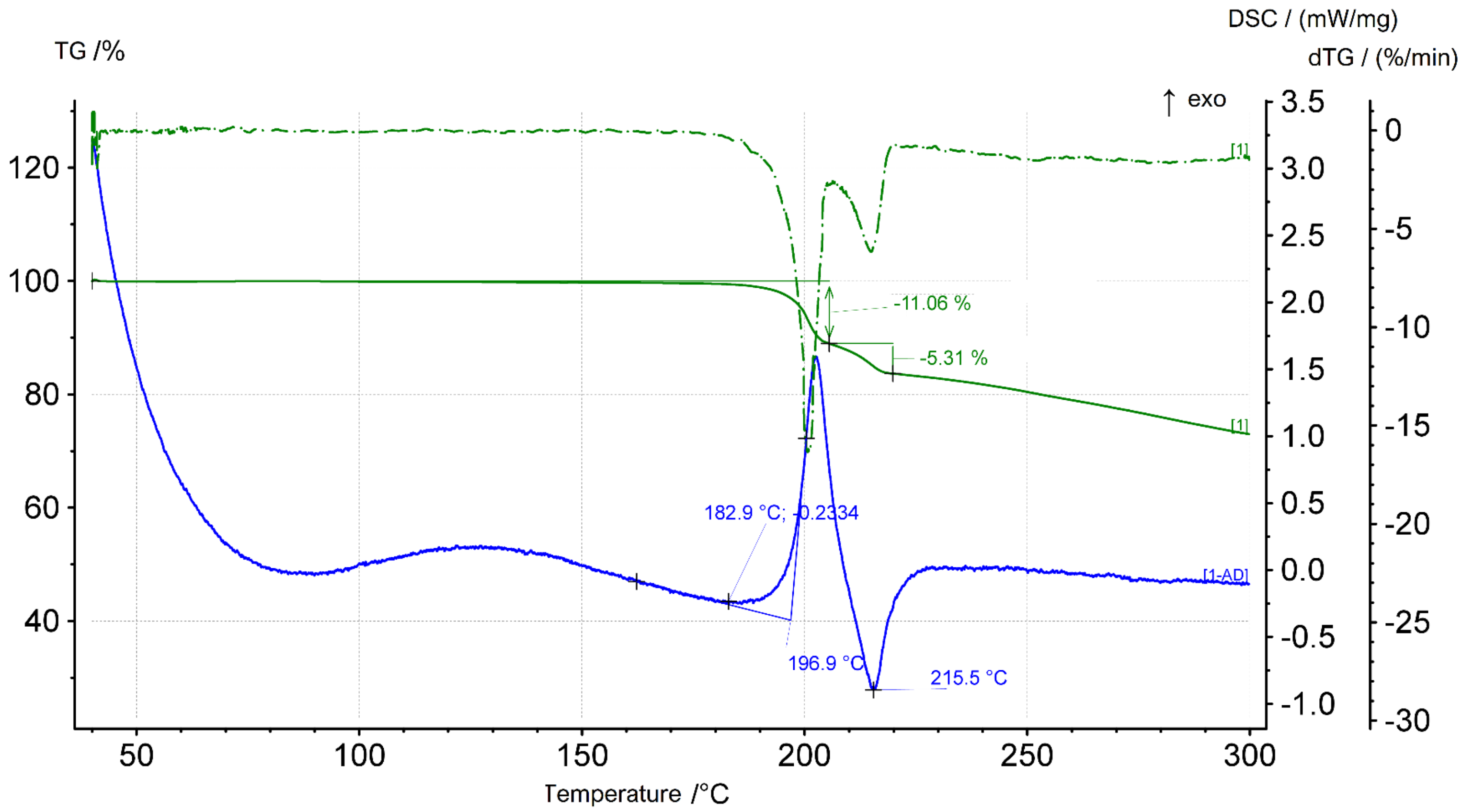

STA plot of thermolysis of APBTT **1f**. Blue solid curve: differential scanning calorimetry (DSC); green solid curve: thermogravimetry (TG); greed dashed curve: derivative thermogravimetry (dTG); heating rate: 10 K/min; argon atmosphere.

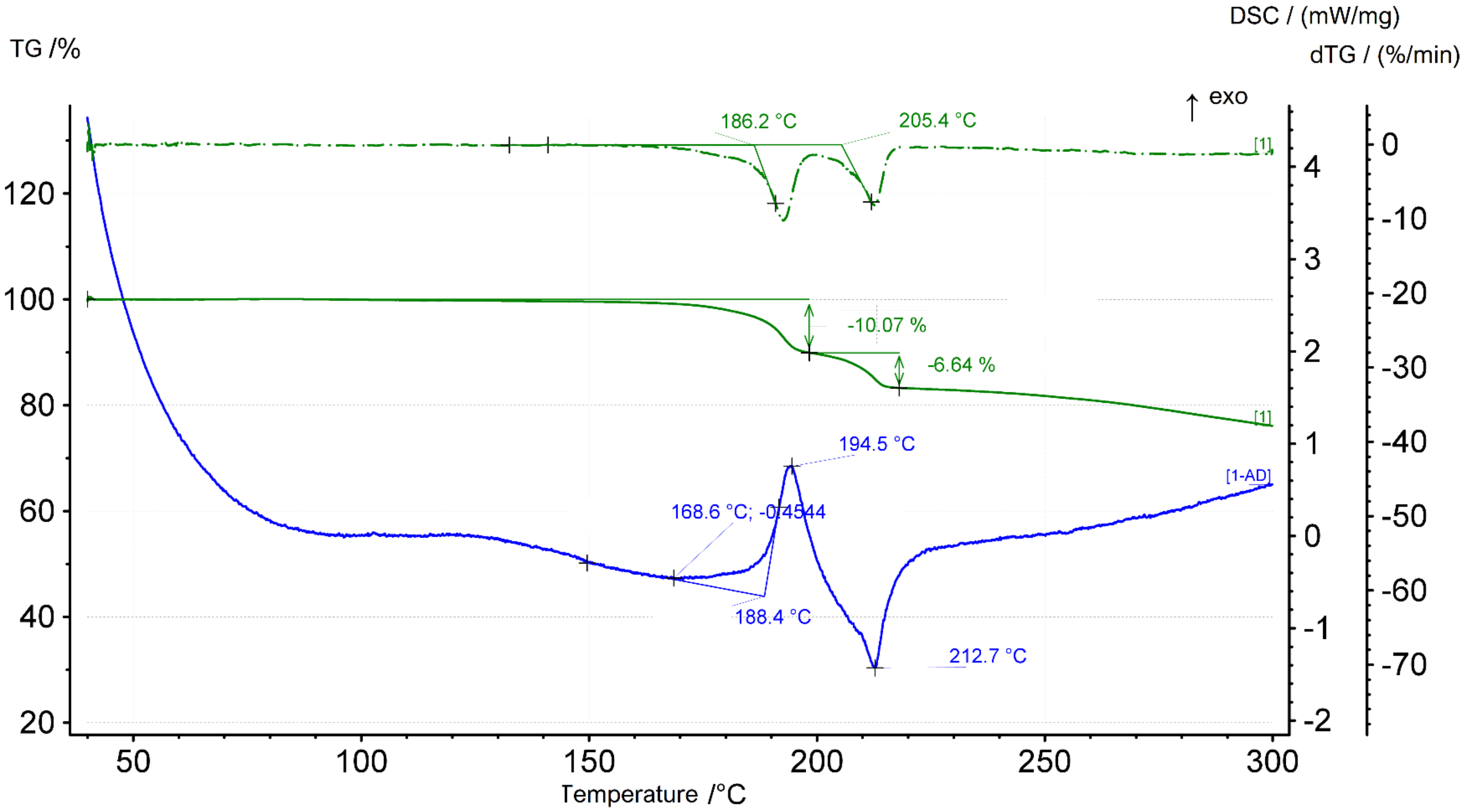

STA plot of thermolysis of APBTT **1g**. Blue solid curve: differential scanning calorimetry (DSC); green solid curve: thermogravimetry (TG); greed dashed curve: derivative thermogravimetry (dTG); heating rate: 10 K/min; argon atmosphere.

STA for compound **4** is given in the main manuscript as Figure 3.

Calculated total electronic energies (E, in Hartree), enthalpies (H, in Hartree), Gibbs free energies (G, in Hartree), and entropies (S, cal/mol•K) for optimized equilibrium model structures.

Model structure	E	H	G	S
C≡O	-113.270735586	-113.262253	-113.284685	47.214
**1a**	-1444.70735195	-1444.455575	-1444.525734	147.663
**2a**	-2436.35209507	-2435.892670	-2435.989048	202.845
**4**	-1331.44520780	-1331.204445	-1331.267874	133.496
**I1**	-1331.40493977	-1331.165836	-1331.232877	141.101
**3a**	-1218.13873072	-1217.911136	-1217.972731	129.637
**I2**	-2436.34498787	-2435.885574	-2435.981547	201.991
**I3**	-2662.86833777	-2662.386369	-2662.491404	221.064
**I4**	-2662.88828775	-2662.405061	-2662.511852	224.762
TS_**1a**to**4**	-1444.67329159	-1444.422782	-1444.487602	136.427
TS_**1a**to**I1**	-1444.65245589	-1444.404343	-1444.475055	148.825
TS_**4**to**3a**	-1331.39047748	-1331.153768	-1331.219893	139.174
TS_**I1**to**3a**	-1331.29957001	-1331.064020	-1331.135054	149.503

HPLC data for thermal decomposition of compounds **1a** and **4**

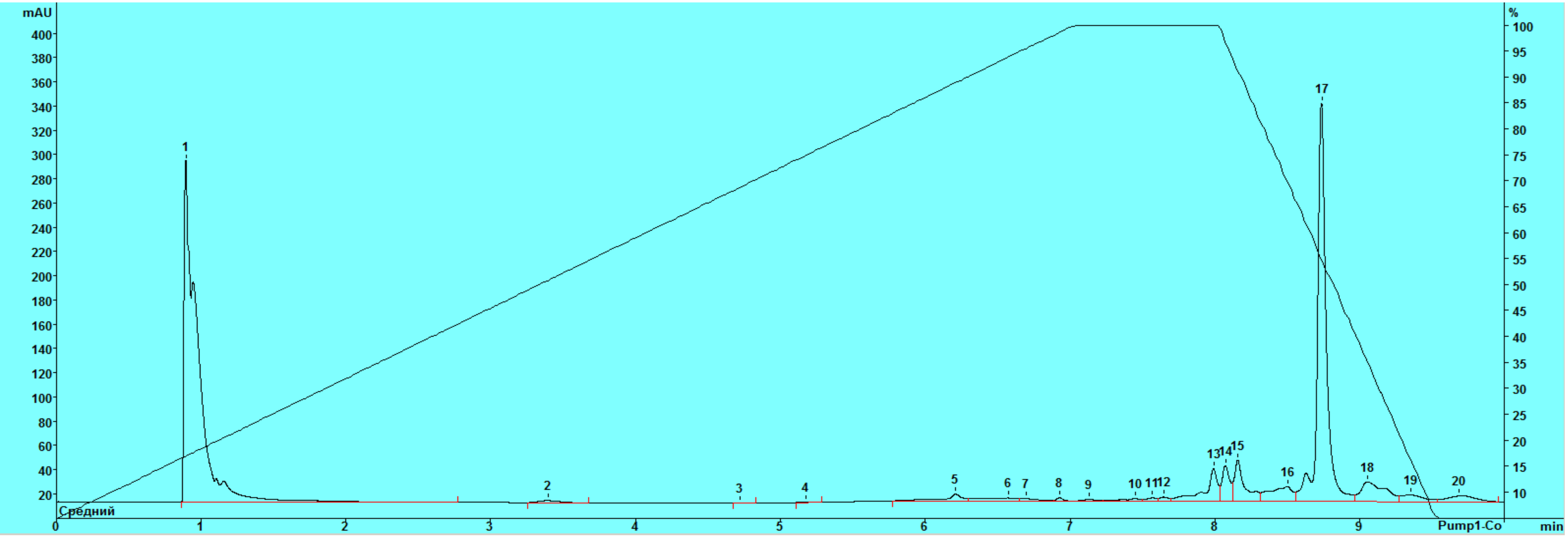

HPLC-UV chromatogram of compound **1a** after measuring of m.p. in a capillary. Peak 17 corresponds to product **2a**. UV signal is given as an average value (λ 210–750 nm).

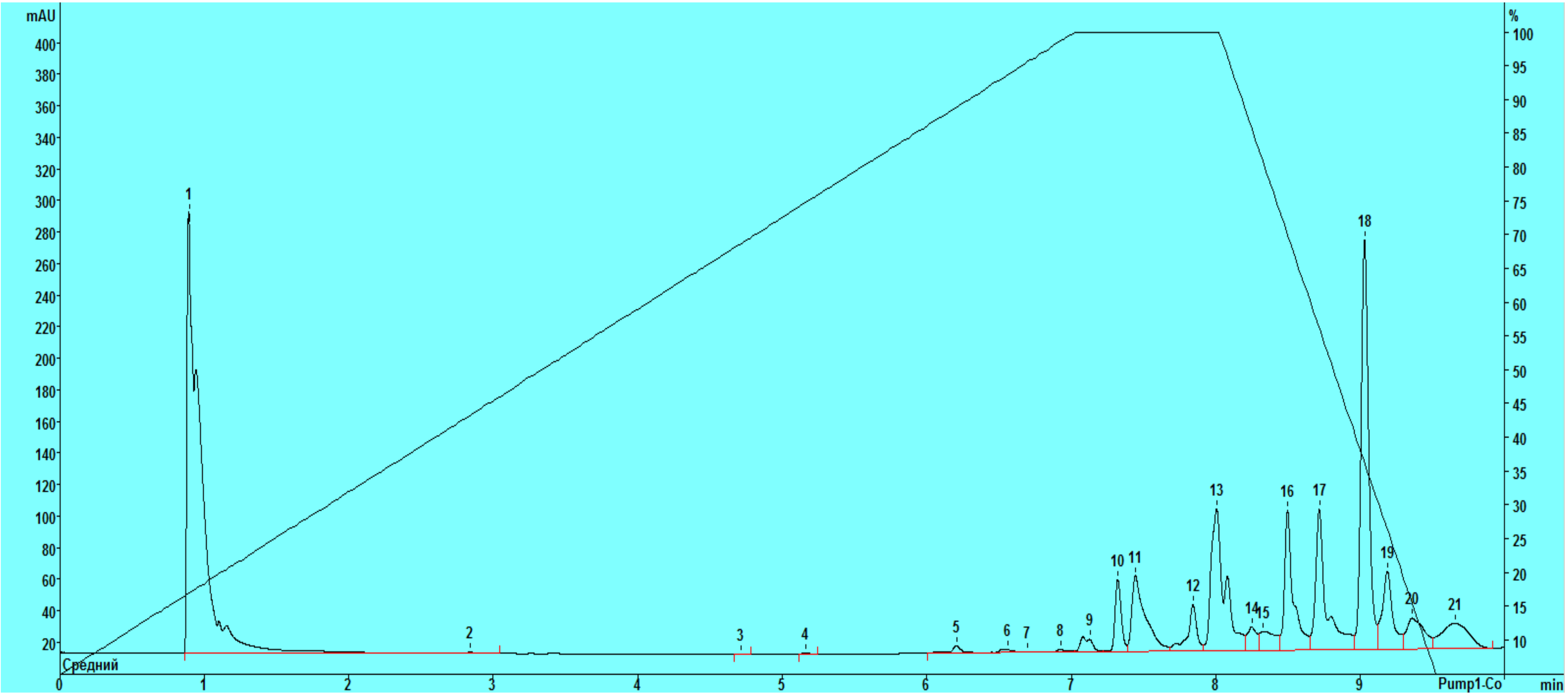

HPLC-UV chromatogram of compound **4** after measuring of m.p. in a capillary. Peak 17 corresponds to product **2a**. UV signal is given as an average value (λ 210–750 nm).

Gradient elution time program:

#	Time, hh : mm : ss	Flow, mL/min	Conc. A, %	Conc. B, %
0	00 : 00 : 00	1.5	95	5
1	00 : 07 : 00	1.5	0	100
2	00 : 08 : 00	1.5	0	100
3	00 : 09 : 30	1.5	95	5
4	00 : 10 : 00	1.5	95	5

A – water, B – acetonitrile.

ORTEP images of X-ray crystal structures of compounds **2a, f**

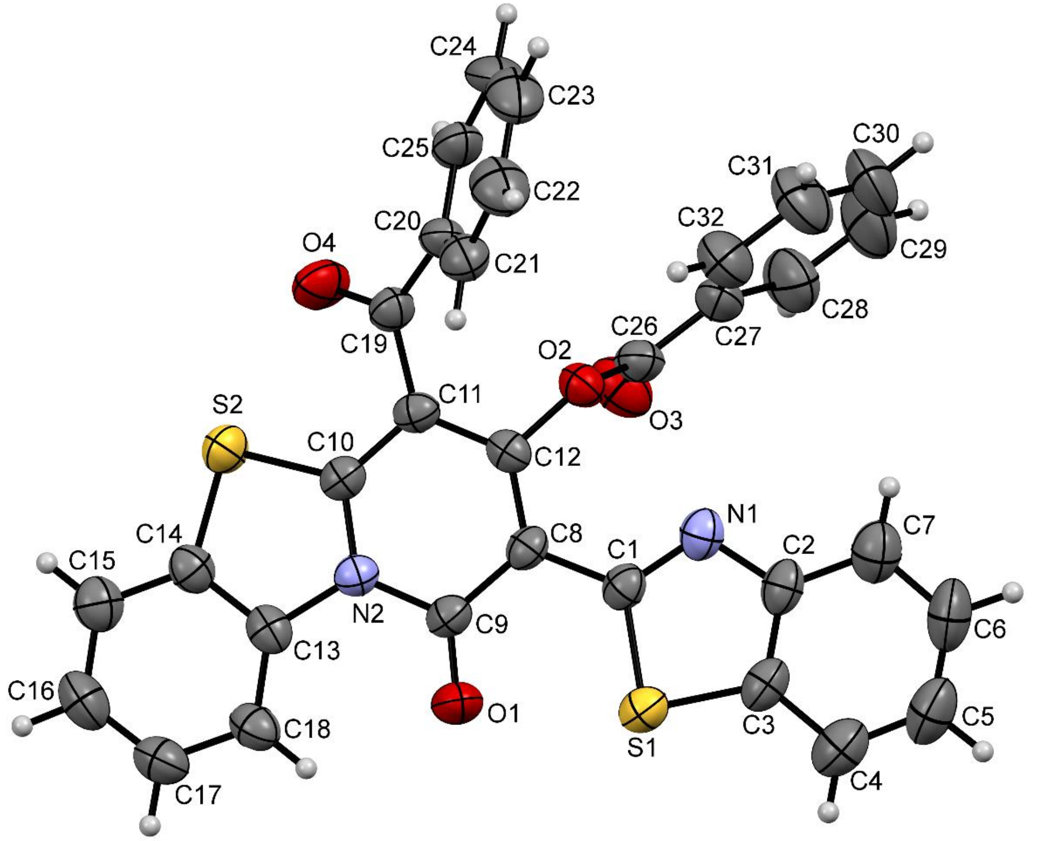

*Molecular structure of compound **2a** showing 30% probability amplitude displacement ellipsoids (CCDC* 2277018*).*

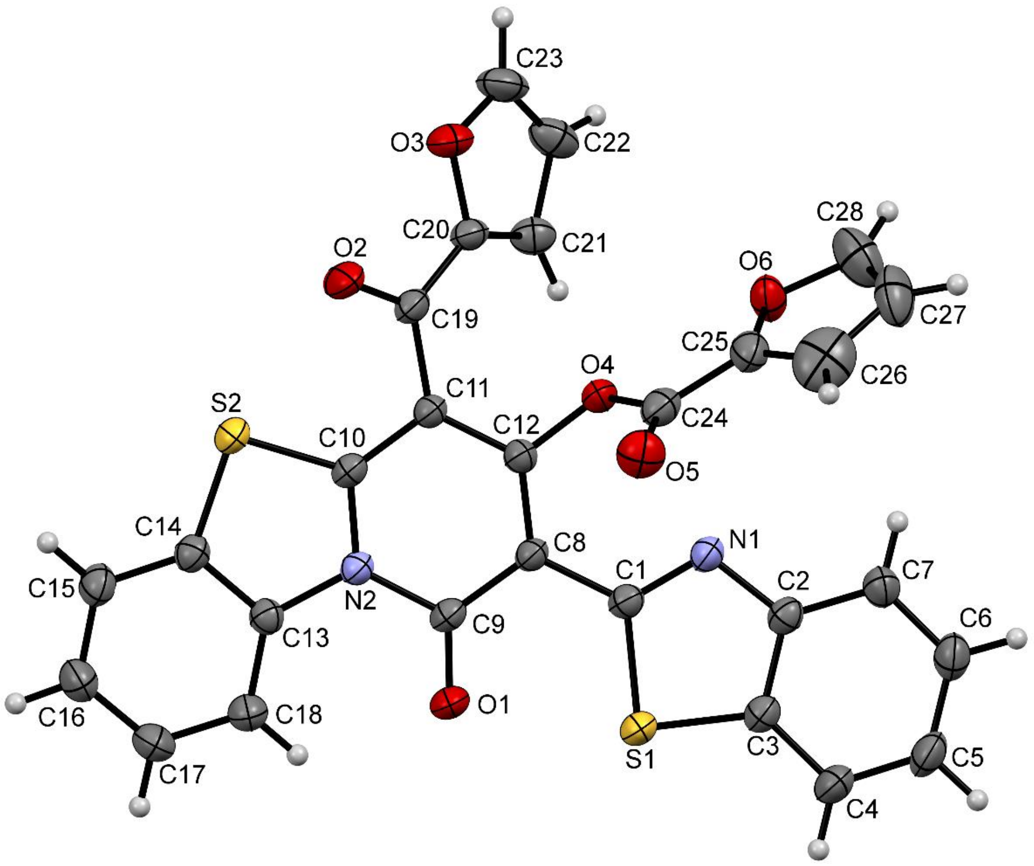

Molecular structure of compound ***2f*** *showing 30% probability amplitude displacement ellipsoids (CCDC* 2277017*).*
